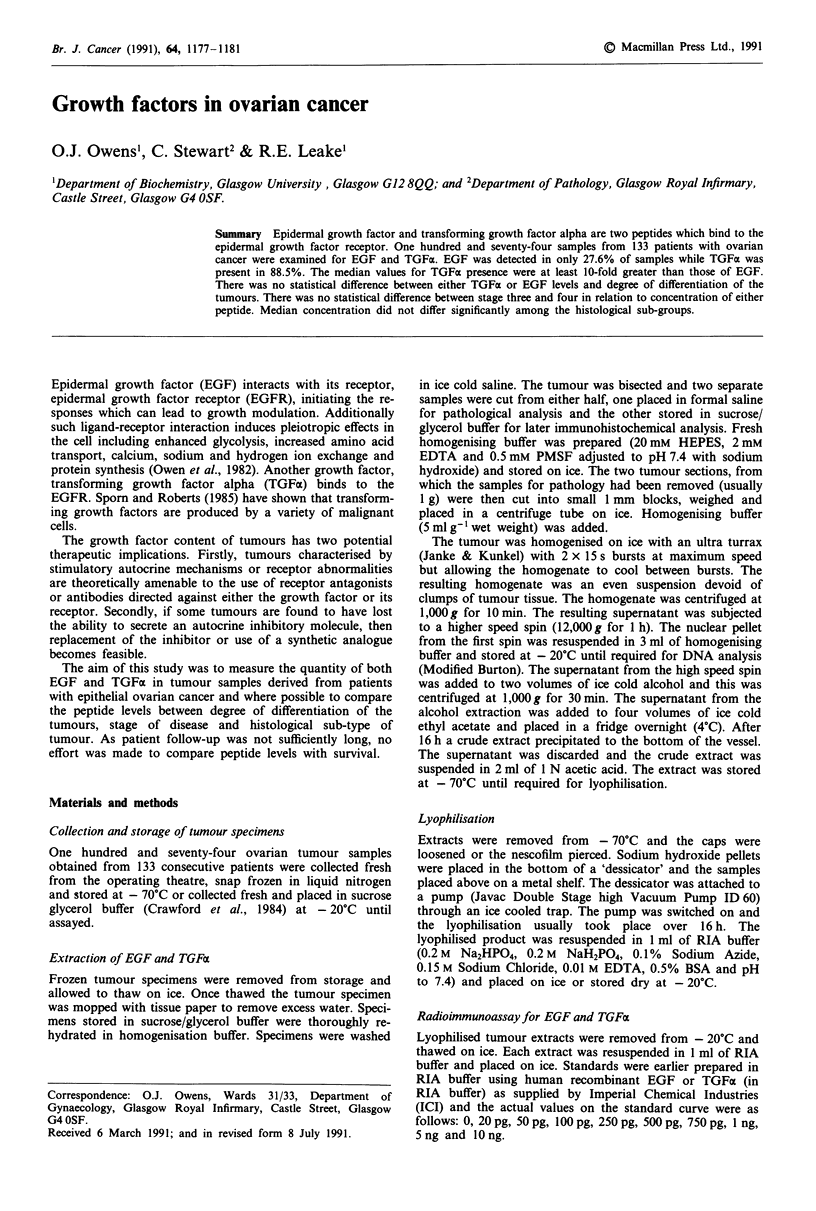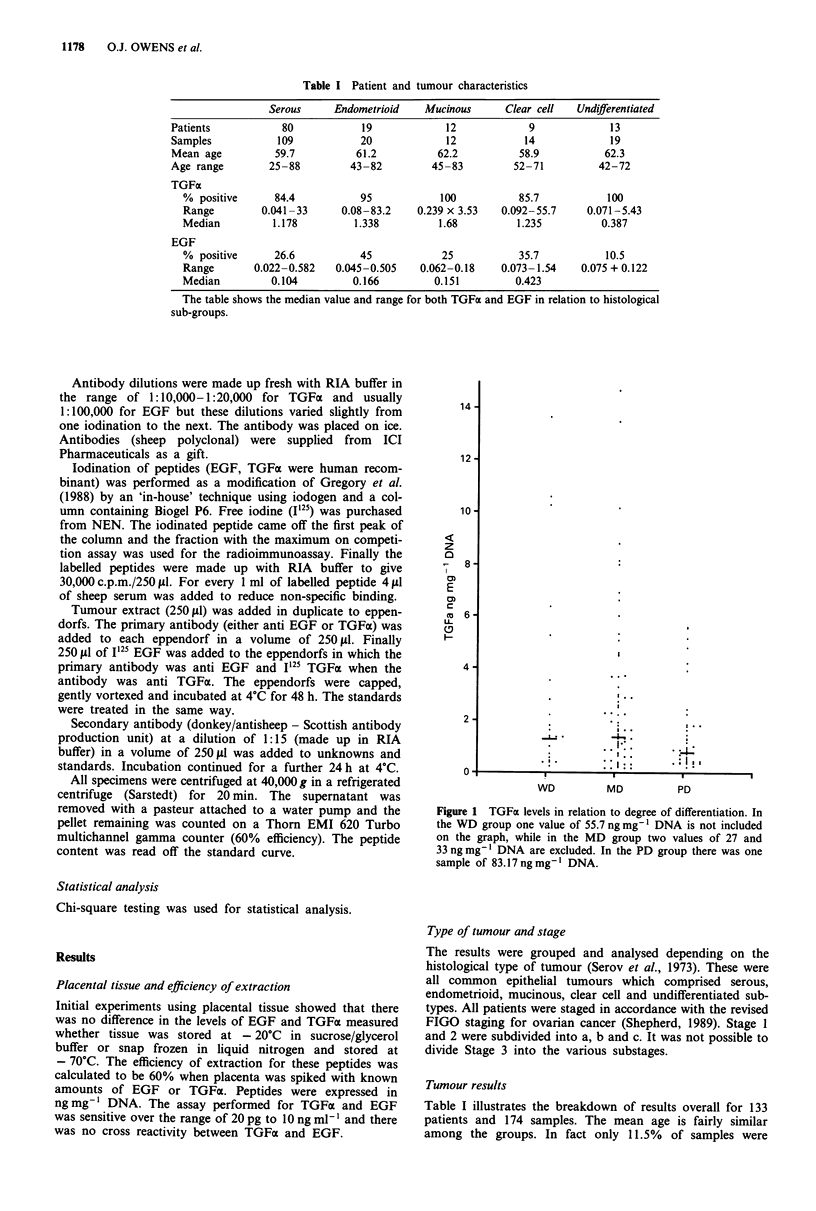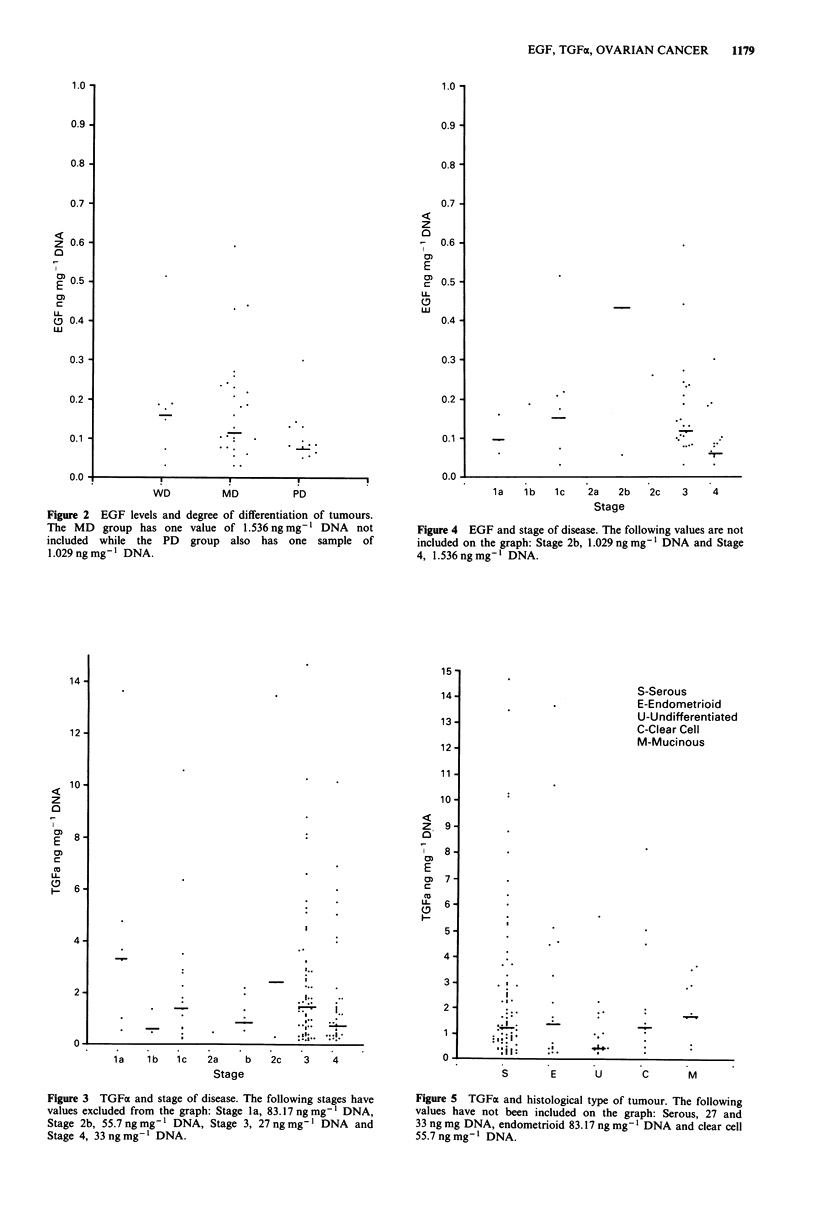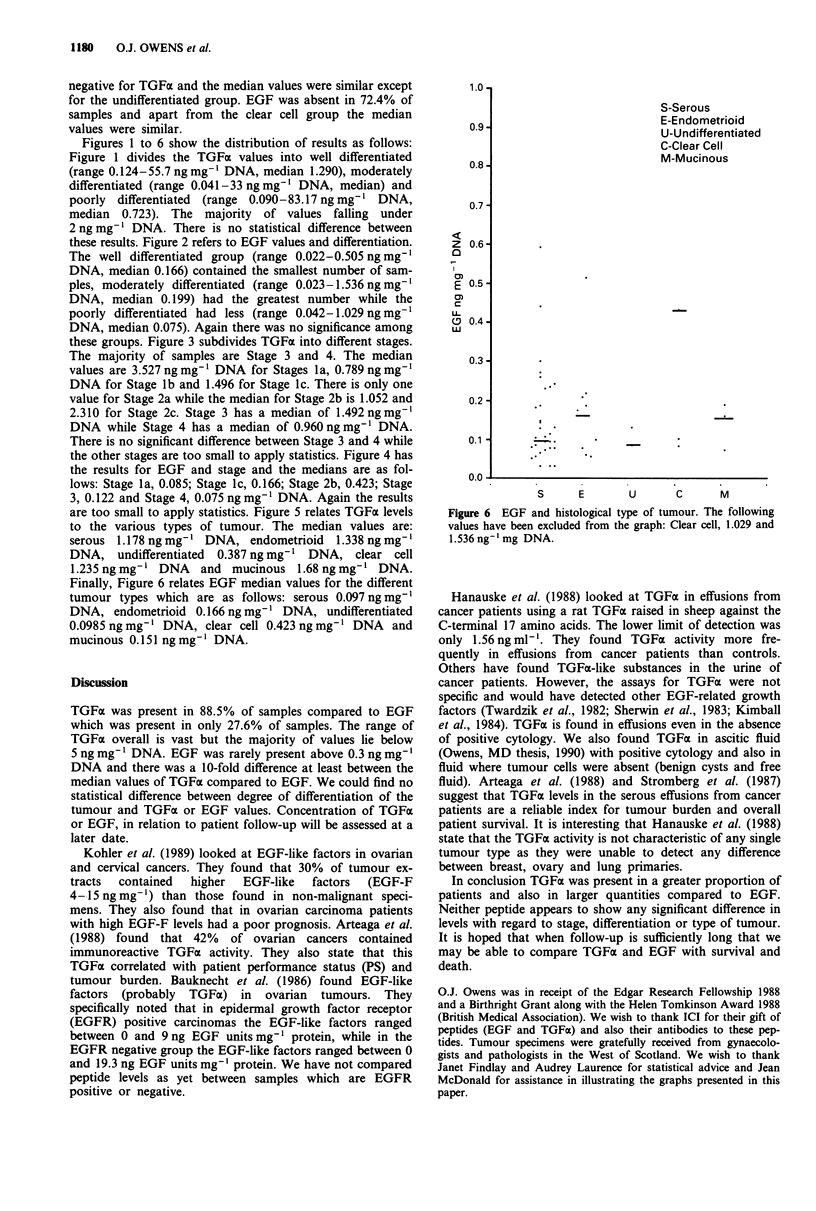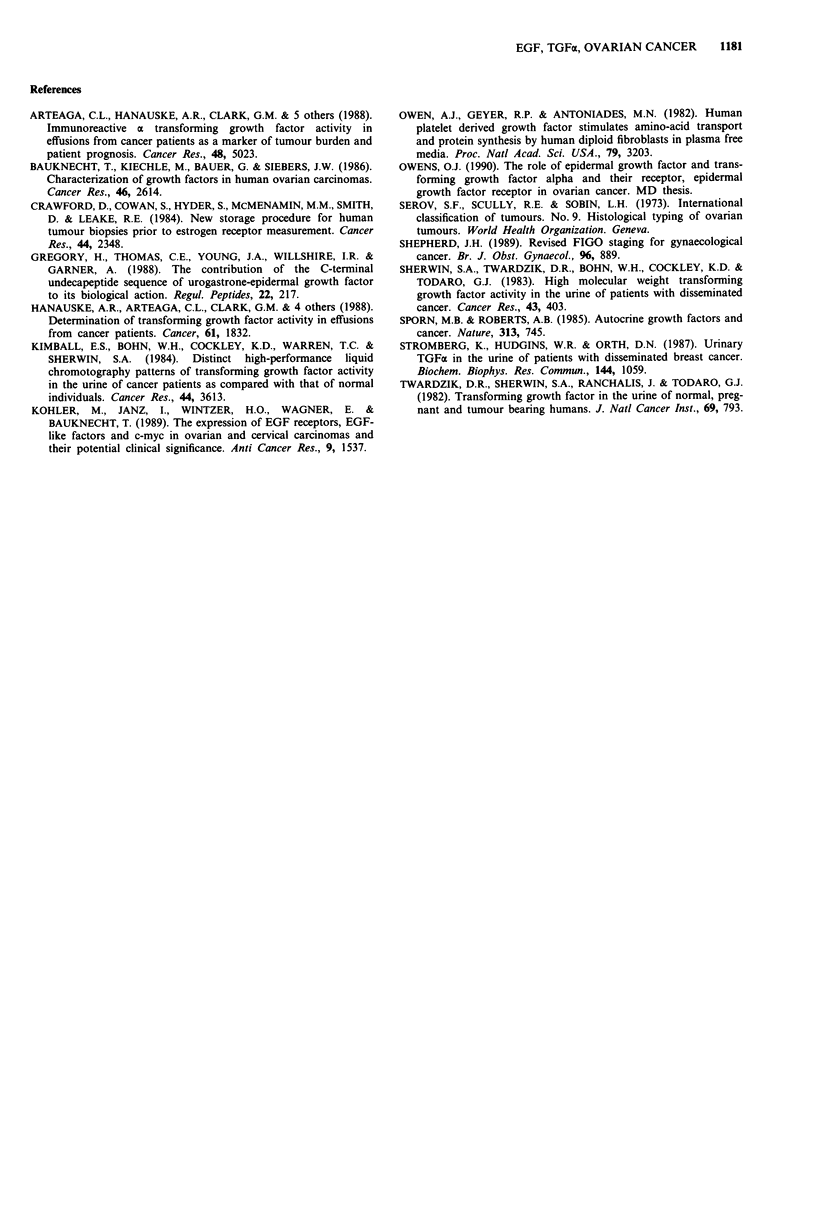# Growth factors in ovarian cancer.

**DOI:** 10.1038/bjc.1991.486

**Published:** 1991-12

**Authors:** O. J. Owens, C. Stewart, R. E. Leake

**Affiliations:** Department of Biochemistry, Glasgow University.

## Abstract

Epidermal growth factor and transforming growth factor alpha are two peptides which bind to the epidermal growth factor receptor. One hundred and seventy-four samples from 133 patients with ovarian cancer were examined for EGF and TGF alpha. EGF was detected in only 27.6% of samples while TGF alpha was present in 88.5%. The median values for TGF alpha presence were at least 10-fold greater than those of EGF. There was no statistical difference between either TGF alpha or EGF levels and degree of differentiation of the tumours. There was no statistical difference between stage three and four in relation to concentration of either peptide. Median concentration did not differ significantly among the histological sub-groups.


*Br. J. Cancer* (1991), **64**, 1177–1181 © Macmillan Press Ltd., 1991

### Growth factors in ovarian cancer

O.J. Owens[1], C. Stewart[2] & R.E. Leake[1]

[1]*Department of Biochemistry, Glasgow University, Glasgow G12 8QQ; and* [2]*Department of Pathology, Glasgow Royal Infirmary, Castle Street, Glasgow G4 0SF.*

**Summary** Epidermal growth factor and transforming growth factor alpha are two peptides which bind to the epidermal growth factor receptor. One hundred and seventy-four samples from 133 patients with ovarian cancer were examined for EGF and TGFα. EGF was detected in only 27.6% of samples while TGFα was present in 88.5%. The median values for TGFα presence were at least 10-fold greater than those of EGF. There was no statistical difference between either TGFα or EGF levels and degree of differentiation of the tumours. There was no statistical difference between stage three and four in relation to concentration of either peptide. Median concentration did not differ significantly among the histological sub-groups.

Epidermal growth factor (EGF) interacts with its receptor, epidermal growth factor receptor (EGFR), initiating the responses which can lead to growth modulation. Additionally such ligand-receptor interaction induces pleiotropic effects in the cell including enhanced glycolysis, increased amino acid transport, calcium, sodium and hydrogen ion exchange and protein synthesis (Owen *et al.*, 1982). Another growth factor, transforming growth factor alpha (TGFα) binds to the EGFR. Sporn and Roberts (1985) have shown that transforming growth factors are produced by a variety of malignant cells.

The growth factor content of tumours has two potential therapeutic implications. Firstly, tumours characterised by stimulatory autocrine mechanisms or receptor abnormalities are theoretically amenable to the use of receptor antagonists or antibodies directed against either the growth factor or its receptor. Secondly, if some tumours are found to have lost the ability to secrete an autocrine inhibitory molecule, then replacement of the inhibitor or use of a synthetic analogue becomes feasible.

The aim of this study was to measure the quantity of both EGF and TGFα in tumour samples derived from patients with epithelial ovarian cancer and where possible to compare the peptide levels between degree of differentiation of the tumours, stage of disease and histological sub-type of tumour. As patient follow-up was not sufficiently long, no effort was made to compare peptide levels with survival.

### Materials and methods

### *Collection and storage of tumour specimens*

One hundred and seventy-four ovarian tumour samples obtained from 133 consecutive patients were collected fresh from the operating theatre, snap frozen in liquid nitrogen and stored at − 70°C or collected fresh and placed in sucrose glycerol buffer (Crawford *et al.*, 1984) at − 20°C until assayed.

### *Extraction of EGF and TGFα*

Frozen tumour specimens were removed from storage and allowed to thaw on ice. Once thawed the tumour specimen was mopped with tissue paper to remove excess water. Specimens stored in sucrose/glycerol buffer were thoroughly rehydrated in homogenisation buffer. Specimens were washed in ice cold saline. The tumour was bisected and two separate samples were cut from either half, one placed in formal saline for pathological analysis and the other stored in sucrose/glycerol buffer for later immunohistochemical analysis. Fresh homogenising buffer was prepared (20 mM HEPES, 2 mM EDTA and 0.5 mM PMSF adjusted to pH 7.4 with sodium hydroxide) and stored on ice. The two tumour sections, from which the samples for pathology had been removed (usually 1 g) were then cut into small 1 mm blocks, weighed and placed in a centrifuge tube on ice. Homogenising buffer (5 ml $g^{-1}$ wet weight) was added.

The tumour was homogenised on ice with an ultra turrax (Janke & Kunkel) with 2 × 15 s bursts at maximum speed but allowing the homogenate to cool between bursts. The resulting homogenate was an even suspension devoid of clumps of tumour tissue. The homogenate was centrifuged at 1,000 *g* for 10 min. The resulting supernatant was subjected to a higher speed spin (12,000 *g* for 1 h). The nuclear pellet from the first spin was resuspended in 3 ml of homogenising buffer and stored at − 20°C until required for DNA analysis (Modified Burton). The supernatant from the high speed spin was added to two volumes of ice cold alcohol and this was centrifuged at 1,000 *g* for 30 min. The supernatant from the alcohol extraction was added to four volumes of ice cold ethyl acetate and placed in a fridge overnight (4°C). After 16 h a crude extract precipitated to the bottom of the vessel. The supernatant was discarded and the crude extract was suspended in 2 ml of 1 N acetic acid. The extract was stored at − 70°C until required for lyophilisation.

### *Lyophilisation*

Extracts were removed from − 70°C and the caps were loosened or the nescofilm pierced. Sodium hydroxide pellets were placed in the bottom of a 'dessicator' and the samples placed above on a metal shelf. The dessicator was attached to a pump (Javac Double Stage high Vacuum Pump ID 60) through an ice cooled trap. The pump was switched on and the lyophilisation usually took place over 16 h. The lyophilised product was resuspended in 1 ml of RIA buffer (0.2 M $Na_2HPO_4$, 0.2 M $NaH_2PO_4$, 0.1% Sodium Azide, 0.15 M Sodium Chloride, 0.01 M EDTA, 0.5% BSA and pH to 7.4) and placed on ice or stored dry at − 20°C.

### *Radioimmunoassay for EGF and TGFα*

Lyophilised tumour extracts were removed from − 20°C and thawed on ice. Each extract was resuspended in 1 ml of RIA buffer and placed on ice. Standards were earlier prepared in RIA buffer using human recombinant EGF or TGFα (in RIA buffer) as supplied by Imperial Chemical Industries (ICI) and the actual values on the standard curve were as follows: 0, 20 pg, 50 pg, 100 pg, 250 pg, 500 pg, 750 pg, 1 ng, 5 ng and 10 ng.

Correspondence: O.J. Owens, Wards 31/33, Department of Gynaecology, Glasgow Royal Infirmary, Castle Street, Glasgow G4 0SF.
Received 6 March 1991; and in revised form 8 July 1991.

**Table I** Patient and tumour characteristics

| | *Serous* | *Endometrioid* | *Mucinous* | *Clear cell* | *Undifferentiated* |
|---|---|---|---|---|---|
| Patients | 80 | 19 | 12 | 9 | 13 |
| Samples | 109 | 20 | 12 | 14 | 19 |
| Mean age | 59.7 | 61.2 | 62.2 | 58.9 | 62.3 |
| Age range | 25–88 | 43–82 | 45–83 | 52–71 | 42–72 |
| TGFα | | | | | |
| % positive | 84.4 | 95 | 100 | 85.7 | 100 |
| Range | 0.041–33 | 0.08–83.2 | 0.239 x 3.53 | 0.092–55.7 | 0.071–5.43 |
| Median | 1.178 | 1.338 | 1.68 | 1.235 | 0.387 |
| EGF | | | | | |
| % positive | 26.6 | 45 | 25 | 35.7 | 10.5 |
| Range | 0.022–0.582 | 0.045–0.505 | 0.062–0.18 | 0.073–1.54 | 0.075 + 0.122 |
| Median | 0.104 | 0.166 | 0.151 | 0.423 | |

The table shows the median value and range for both TGFα and EGF in relation to histological sub-groups.

Antibody dilutions were made up fresh with RIA buffer in the range of 1:10,000–1:20,000 for TGFα and usually 1:100,000 for EGF but these dilutions varied slightly from one iodination to the next. The antibody was placed on ice. Antibodies (sheep polyclonal) were supplied from ICI Pharmaceuticals as a gift.

Iodination of peptides (EGF, TGFα were human recombinant) was performed as a modification of Gregory *et al.* (1988) by an 'in-house' technique using iodogen and a column containing Biogel P6. Free iodine ($I^{125}$) was purchased from NEN. The iodinated peptide came off the first peak of the column and the fraction with the maximum on competition assay was used for the radioimmunoassay. Finally the labelled peptides were made up with RIA buffer to give 30,000 c.p.m./250 µl. For every 1 ml of labelled peptide 4 µl of sheep serum was added to reduce non-specific binding.

Tumour extract (250 µl) was added in duplicate to eppendorfs. The primary antibody (either anti EGF or TGFα) was added to each eppendorf in a volume of 250 µl. Finally 250 µl of $I^{125}$ EGF was added to the eppendorfs in which the primary antibody was anti EGF and $I^{125}$ TGFα when the antibody was anti TGFα. The eppendorfs were capped, gently vortexed and incubated at 4°C for 48 h. The standards were treated in the same way.

Secondary antibody (donkey/antisheep – Scottish antibody production unit) at a dilution of 1:15 (made up in RIA buffer) in a volume of 250 µl was added to unknowns and standards. Incubation continued for a further 24 h at 4°C.

All specimens were centrifuged at 40,000 *g* in a refrigerated centrifuge (Sarstedt) for 20 min. The supernatant was removed with a pasteur attached to a water pump and the pellet remaining was counted on a Thorn EMI 620 Turbo multichannel gamma counter (60% efficiency). The peptide content was read off the standard curve.

### *Statistical analysis*

Chi-square testing was used for statistical analysis.

### Results

### *Placental tissue and efficiency of extraction*

Initial experiments using placental tissue showed that there was no difference in the levels of EGF and TGFα measured whether tissue was stored at – 20°C in sucrose/glycerol buffer or snap frozen in liquid nitrogen and stored at – 70°C. The efficiency of extraction for these peptides was calculated to be 60% when placenta was spiked with known amounts of EGF or TGFα. Peptides were expressed in ng mg$^{-1}$ DNA. The assay performed for TGFα and EGF was sensitive over the range of 20 pg to 10 ng ml$^{-1}$ and there was no cross reactivity between TGFα and EGF.

**Figure 1** TGFα levels in relation to degree of differentiation. In the WD group one value of 55.7 ng mg$^{-1}$ DNA is not included on the graph, while in the MD group two values of 27 and 33 ng mg$^{-1}$ DNA are excluded. In the PD group there was one sample of 83.17 ng mg$^{-1}$ DNA.

### *Type of tumour and stage*

The results were grouped and analysed depending on the histological type of tumour (Serov *et al.*, 1973). These were all common epithelial tumours which comprised serous, endometrioid, mucinous, clear cell and undifferentiated subtypes. All patients were staged in accordance with the revised FIGO staging for ovarian cancer (Shepherd, 1989). Stage 1 and 2 were subdivided into a, b and c. It was not possible to divide Stage 3 into the various substages.

### *Tumour results*

Table I illustrates the breakdown of results overall for 133 patients and 174 samples. The mean age is fairly similar among the groups. In fact only 11.5% of samples were

**Figure 2** EGF levels and degree of differentiation of tumours. The MD group has one value of 1.536 ng mg$^{-1}$ DNA not included while the PD group also has one sample of 1.029 ng mg$^{-1}$ DNA.

**Figure 4** EGF and stage of disease. The following values are not included on the graph: Stage 2b, 1.029 ng mg$^{-1}$ DNA and Stage 4, 1.536 ng mg$^{-1}$ DNA.

**Figure 3** TGFα and stage of disease. The following stages have values excluded from the graph: Stage 1a, 83.17 ng mg$^{-1}$ DNA, Stage 2b, 55.7 ng mg$^{-1}$ DNA, Stage 3, 27 ng mg$^{-1}$ DNA and Stage 4, 33 ng mg$^{-1}$ DNA.

**Figure 5** TGFα and histological type of tumour. The following values have not been included on the graph: Serous, 27 and 33 ng mg DNA, endometrioid 83.17 ng mg$^{-1}$ DNA and clear cell 55.7 ng mg$^{-1}$ DNA.

negative for TGFα and the median values were similar except for the undifferentiated group. EGF was absent in 72.4% of samples and apart from the clear cell group the median values were similar.

Figures 1 to 6 show the distribution of results as follows: Figure 1 divides the TGFα values into well differentiated (range 0.124–55.7 ng mg$^{-1}$ DNA, median 1.290), moderately differentiated (range 0.041–33 ng mg$^{-1}$ DNA, median) and poorly differentiated (range 0.090–83.17 ng mg$^{-1}$ DNA, median 0.723). The majority of values falling under 2 ng mg$^{-1}$ DNA. There is no statistical difference between these results. Figure 2 refers to EGF values and differentiation. The well differentiated group (range 0.022–0.505 ng mg$^{-1}$ DNA, median 0.166) contained the smallest number of samples, moderately differentiated (range 0.023–1.536 ng mg$^{-1}$ DNA, median 0.199) had the greatest number while the poorly differentiated had less (range 0.042–1.029 ng mg$^{-1}$ DNA, median 0.075). Again there was no significance among these groups. Figure 3 subdivides TGFα into different stages. The majority of samples are Stage 3 and 4. The median values are 3.527 ng mg$^{-1}$ DNA for Stages 1a, 0.789 ng mg$^{-1}$ DNA for Stage 1b and 1.496 for Stage 1c. There is only one value for Stage 2a while the median for Stage 2b is 1.052 and 2.310 for Stage 2c. Stage 3 has a median of 1.492 ng mg$^{-1}$ DNA while Stage 4 has a median of 0.960 ng mg$^{-1}$ DNA. There is no significant difference between Stage 3 and 4 while the other stages are too small to apply statistics. Figure 4 has the results for EGF and stage and the medians are as follows: Stage 1a, 0.085; Stage 1c, 0.166; Stage 2b, 0.423; Stage 3, 0.122 and Stage 4, 0.075 ng mg$^{-1}$ DNA. Again the results are too small to apply statistics. Figure 5 relates TGFα levels to the various types of tumour. The median values are: serous 1.178 ng mg$^{-1}$ DNA, endometrioid 1.338 ng mg$^{-1}$ DNA, undifferentiated 0.387 ng mg$^{-1}$ DNA, clear cell 1.235 ng mg$^{-1}$ DNA and mucinous 1.68 ng mg$^{-1}$ DNA. Finally, Figure 6 relates EGF median values for the different tumour types which are as follows: serous 0.097 ng mg$^{-1}$ DNA, endometrioid 0.166 ng mg$^{-1}$ DNA, undifferentiated 0.0985 ng mg$^{-1}$ DNA, clear cell 0.423 ng mg$^{-1}$ DNA and mucinous 0.151 ng mg$^{-1}$ DNA.

**Figure 6** EGF and histological type of tumour. The following values have been excluded from the graph: Clear cell, 1.029 and 1.536 ng$^{-1}$ mg DNA.

### Discussion

TGFα was present in 88.5% of samples compared to EGF which was present in only 27.6% of samples. The range of TGFα overall is vast but the majority of values lie below 5 ng mg$^{-1}$ DNA. EGF was rarely present above 0.3 ng mg$^{-1}$ DNA and there was a 10-fold difference at least between the median values of TGFα compared to EGF. We could find no statistical difference between degree of differentiation of the tumour and TGFα or EGF values. Concentration of TGFα or EGF, in relation to patient follow-up will be assessed at a later date.

Kohler *et al.* (1989) looked at EGF-like factors in ovarian and cervical cancers. They found that 30% of tumour extracts contained higher EGF-like factors (EGF-F 4–15 ng mg$^{-1}$) than those found in non-malignant specimens. They also found that in ovarian carcinoma patients with high EGF-F levels had a poor prognosis. Arteaga *et al.* (1988) found that 42% of ovarian cancers contained immunoreactive TGFα activity. They also state that this TGFα correlated with patient performance status (PS) and tumour burden. Bauknecht *et al.* (1986) found EGF-like factors (probably TGFα) in ovarian tumours. They specifically noted that in epidermal growth factor receptor (EGFR) positive carcinomas the EGF-like factors ranged between 0 and 9 ng EGF units mg$^{-1}$ protein, while in the EGFR negative group the EGF-like factors ranged between 0 and 19.3 ng EGF units mg$^{-1}$ protein. We have not compared peptide levels as yet between samples which are EGFR positive or negative.

Hanauske *et al.* (1988) looked at TGFα in effusions from cancer patients using a rat TGFα raised in sheep against the C-terminal 17 amino acids. The lower limit of detection was only 1.56 ng ml$^{-1}$. They found TGFα activity more frequently in effusions from cancer patients than controls. Others have found TGFα-like substances in the urine of cancer patients. However, the assays for TGFα were not specific and would have detected other EGF-related growth factors (Twardzik *et al.*, 1982; Sherwin *et al.*, 1983; Kimball *et al.*, 1984). TGFα is found in effusions even in the absence of positive cytology. We also found TGFα in ascitic fluid (Owens, MD thesis, 1990) with positive cytology and also in fluid where tumour cells were absent (benign cysts and free fluid). Arteaga *et al.* (1988) and Stromberg *et al.* (1987) suggest that TGFα levels in the serous effusions from cancer patients are a reliable index for tumour burden and overall patient survival. It is interesting that Hanauske *et al.* (1988) state that the TGFα activity is not characteristic of any single tumour type as they were unable to detect any difference between breast, ovary and lung primaries.

In conclusion TGFα was present in a greater proportion of patients and also in larger quantities compared to EGF. Neither peptide appears to show any significant difference in levels with regard to stage, differentiation or type of tumour. It is hoped that when follow-up is sufficiently long that we may be able to compare TGFα and EGF with survival and death.

O.J. Owens was in receipt of the Edgar Research Fellowship 1988 and a Birthright Grant along with the Helen Tomkinson Award 1988 (British Medical Association). We wish to thank ICI for their gift of peptides (EGF and TGFα) and also their antibodies to these peptides. Tumour specimens were gratefully received from gynaecologists and pathologists in the West of Scotland. We wish to thank Janet Findlay and Audrey Laurence for statistical advice and Jean McDonald for assistance in illustrating the graphs presented in this paper.

### References

ARTEAGA, C.L., HANAUSKE, A.R., CLARK, G.M. & 5 others (1988). Immunoreactive α transforming growth factor activity in effusions from cancer patients as a marker of tumour burden and patient prognosis. *Cancer Res.*, **48**, 5023.

BAUKNECHT, T., KIECHLE, M., BAUER, G. & SIEBERS, J.W. (1986). Characterization of growth factors in human ovarian carcinomas. *Cancer Res.*, **46**, 2614.

CRAWFORD, D., COWAN, S., HYDER, S., MCMENAMIN, M.M., SMITH, D. & LEAKE, R.E. (1984). New storage procedure for human tumour biopsies prior to estrogen receptor measurement. *Cancer Res.*, **44**, 2348.

GREGORY, H., THOMAS, C.E., YOUNG, J.A., WILLSHIRE, I.R. & GARNER, A. (1988). The contribution of the C-terminal undecapeptide sequence of urogastrone-epidermal growth factor to its biological action. *Regul. Peptides*, **22**, 217.

HANAUSKE, A.R., ARTEAGA, C.L., CLARK, G.M. & 4 others (1988). Determination of transforming growth factor activity in effusions from cancer patients. *Cancer*, **61**, 1832.

KIMBALL, E.S., BOHN, W.H., COCKLEY, K.D., WARREN, T.C. & SHERWIN, S.A. (1984). Distinct high-performance liquid chromotography patterns of transforming growth factor activity in the urine of cancer patients as compared with that of normal individuals. *Cancer Res.*, **44**, 3613.

KOHLER, M., JANZ, I., WINTZER, H.O., WAGNER, E. & BAUKNECHT, T. (1989). The expression of EGF receptors, EGF-like factors and c-myc in ovarian and cervical carcinomas and their potential clinical significance. *Anti Cancer Res.*, **9**, 1537.

OWEN, A.J., GEYER, R.P. & ANTONIADES, M.N. (1982). Human platelet derived growth factor stimulates amino-acid transport and protein synthesis by human diploid fibroblasts in plasma free media. *Proc. Natl Acad. Sci. USA.*, **79**, 3203.

OWENS, O.J. (1990). The role of epidermal growth factor and transforming growth factor alpha and their receptor, epidermal growth factor receptor in ovarian cancer. MD thesis.

SEROV, S.F., SCULLY, R.E. & SOBIN, L.H. (1973). International classification of tumours. No. 9. Histological typing of ovarian tumours. *World Health Organization. Geneva.*

SHEPHERD, J.H. (1989). Revised FIGO staging for gynaecological cancer. *Br. J. Obst. Gynaecol.*, **96**, 889.

SHERWIN, S.A., TWARDZIK, D.R., BOHN, W.H., COCKLEY, K.D. & TODARO, G.J. (1983). High molecular weight transforming growth factor activity in the urine of patients with disseminated cancer. *Cancer Res.*, **43**, 403.

SPORN, M.B. & ROBERTS, A.B. (1985). Autocrine growth factors and cancer. *Nature*, **313**, 745.

STROMBERG, K., HUDGINS, W.R. & ORTH, D.N. (1987). Urinary TGFα in the urine of patients with disseminated breast cancer. *Biochem. Biophys. Res. Commun.*, **144**, 1059.

TWARDZIK, D.R., SHERWIN, S.A., RANCHALIS, J. & TODARO, G.J. (1982). Transforming growth factor in the urine of normal, pregnant and tumour bearing humans. *J. Natl Cancer Inst.*, **69**, 793.