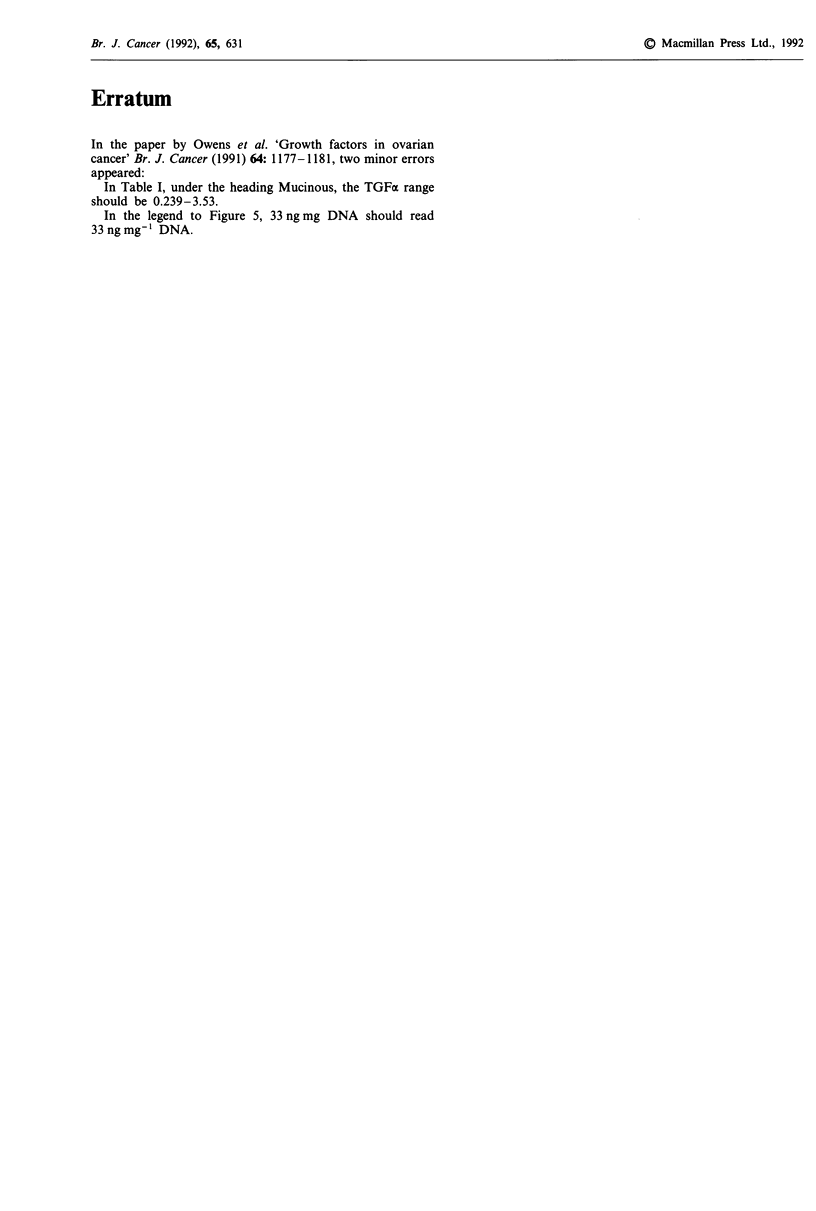# Erratum

**Published:** 1992-04

**Authors:** 


 © Macmillan Press Ltd., 1992

### Erratum

In the paper by Owens *et al.* 'Growth factors in ovarian cancer' *Br. J. Cancer* (1991) **64:** 1177–1181, two minor errors appeared:

In Table I, under the heading Mucinous, the TGFα range should be 0.239–3.53.

In the legend to Figure 5, 33 ng mg DNA should read 33 ng $mg^{-1}$ DNA.